# Polymorphisms and Pharmacogenomics of *NQO2*: The Past and the Future

**DOI:** 10.3390/genes15010087

**Published:** 2024-01-10

**Authors:** Elzbieta Janda, Jean A. Boutin, Carlo De Lorenzo, Mariamena Arbitrio

**Affiliations:** 1 Laboratory of Cellular and Molecular Toxicology, Department of Health Science, University “Magna Græcia” of Catanzaro, 88100 Catanzaro, Italy; 2 Laboratory of Neuroendocrine Endocrine and Germinal Differentiation and Communication (NorDiC), Université de Rouen Normandie, INSERM, UMR 1239, 76000 Rouen, France; ja.boutin.pro@gmail.com; 3 Institute for Biomedical Research and Innovation (IRIB), National Research Council of Italy (CNR), 88100 Catanzaro, Italy

**Keywords:** NQO2, QR2, SNP, cancer, pharmacogenomics, neurodegenerative disease

## Abstract

The flavoenzyme N-ribosyldihydronicotinamide (NRH):quinone oxidoreductase 2 (NQO2) catalyzes two-electron reductions of quinones. NQO2 contributes to the metabolism of biogenic and xenobiotic quinones, including a wide range of antitumor drugs, with both toxifying and detoxifying functions. Moreover, NQO2 activity can be inhibited by several compounds, including drugs and phytochemicals such as flavonoids. NQO2 may play important roles that go beyond quinone metabolism and include the regulation of oxidative stress, inflammation, and autophagy, with implications in carcinogenesis and neurodegeneration. *NQO2* is a highly polymorphic gene with several allelic variants, including insertions (I), deletions (D) and single-nucleotide (SNP) polymorphisms located mainly in the promoter, but also in other regulatory regions and exons. This is the first systematic review of the literature reporting on NQO2 gene variants as risk factors in degenerative diseases or drug adverse effects. In particular, hypomorphic 29 bp I alleles have been linked to breast and other solid cancer susceptibility as well as to interindividual variability in response to chemotherapy. On the other hand, hypermorphic polymorphisms were associated with Parkinson’s and Alzheimer’s disease. The I and D promoter variants and other NQO2 polymorphisms may impact cognitive decline, alcoholism and toxicity of several nervous system drugs. Future studies are required to fill several gaps in NQO2 research.

## 1. Introduction

NQO2, also known as NRH:quinone oxidoreductase (EC: 1.10.99.2) (NQO2 EC: 1.10.99.2 should not be confused with ubiquinone oxidoreductase, also known as NQO2 EC: 1.6.5.3, a sub-unit of the mitochondrial complex I) or QR2, is a 26 kDa flavine adenine dinucleotide (FAD)-containing oxidoreductase that catalyzes two-electron reductions of quinones, pseudoquinones and other related electron acceptors. This flavoprotein is a member of the quinone oxidoreductases (QOR) subgroup of the flavodoxin-2 protein family [1]. NQO2 has a structural similarity and sequence homology to another FAD-bound protein, NQO1 (previously known as DT-diaphorase or quinone reductase 1). Both enzymes show partially overlapping substrate specificities and functions in interactions with different drugs, xenobiotics and endogenous compounds [1,2]. However, there are some important differences between these enzymes. In contrast to NQO1, NQO2 is an atypical oxidoreductase since it is unable to recognize classical electron donors, such as NADH nor NAD(P)H. Instead, NQO2 recognizes a series of electron donors that are derived from NADH, such as N-methyldihydronicotinamide (NMH), N-ribosyldihydronicotinamide (NRH), or their synthetic counterpart, N-benzyldihyrdonicotinamide (BNAH). NRH and NMH are biogenic intermediates of NADH metabolism [3], but it is unclear when they become abundant to activate the NQO2 enzymatic function. This peculiar feature of NQO2 led to the concept that NQO2 may also work as an intracellular pseudoenzyme independent of its catalytic activity [4,5]. Nevertheless, NQO2 enzymatic activity has been linked to both detoxifying and toxifying functions in vivo. In fact, the chemical reactions carried by NQO2 involve a “ping-pong” two-electron transfer to quinone substrates [6,7]. Such a reduction of quinones produces variably stable hydroquinones that can be eliminated by conjugation with sulfates and glucuronides by different II-phase drug metabolic enzymes and excreted as conjugates [8,9]. This mechanism was initially proposed as a safe way of reducing quinones without the generation of semiquinones and ROS, but challenged by more recent findings suggesting that NQO2 can produce free radicals as a by-product of spontaneous autooxidation of hydroquinones [10]. NQO2 may work as a toxifying enzyme for its substrates, but it also contributes to the production of free radicals in the presence of compounds that are not NQO2 substrates, by yet unknown mechanisms. This peculiar role of NQO2 was observed in astrocytes treated with neurotoxins such as paraquat, 6-OH-dopamine and MPTP, but not in dopaminergic SH-SY5Y cells [11,12,13,14]. Thus, the current view is that NQO2 has a dual nature, either detoxifying or toxifying depending on the substrate, cell type, expression and activity of other detoxifying enzymes such as UDP-glucuronosyltransferase [1,9]. In addition, the detoxifying or toxic effect of the NQO2 activity might be determined by amino acid sequence differences in polymorphic variants and orthologs as discussed below. Another peculiar feature of NQO2 is that it can be inhibited or interact with a plentitude of compounds. The 30 years of NQO2 research have led to the identification of hundreds of various inhibitors including several biogenic compounds and phytochemicals, as well as synthetic drugs. In this respect, NQO2 has been described as the drug target with the highest hit percentage (29%), defined as the number of hits (positive interactions) divided by the number of compounds tested at each target [15]. Thus, NQO2 presents broad substrate specificities, which are outstanding among other drug metabolism enzymes, characterized by the ability to accommodate many structurally different xenobiotics. These findings support the relevance of NQO2 in pharmacogenomics (PGx) and drug metabolism studies, but also emphasize the need for a better understanding of the biological functions of this protein in health and disease, as discussed in this article.

### 1.1. NQO2 Gene Structure and Its Polymorphic Variants

**Figure 1:**
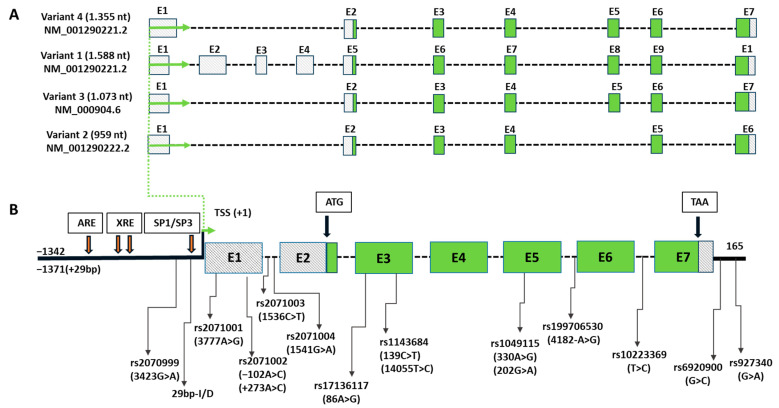
(**A**) Genomic structure of four alternative transcripts of human *NQO2*; (**B**) representative genomic structure of an NQO2 transcript with magnified exons (E1 to E7) and with indicated position of known disease-related *NQO2* polymorphisms (or implicated in drug adverse effects by a statistically significant *p*-value in Cox regression analysis in at least one study). Filled green boxes: coding regions of exons; marked white boxes: 5′-UTR and 3′-UTR; broken black lines: introns; bold black lines: 5′- and 3′-flanking genomic regulatory regions; green arrows: transcription start; ARE, antioxidant response element; SP1/SP3 binding sites; TSS, transcription start site; XRE, xenobiotic response element.

The human *NQO2* gene is located on the short arm of the chromosome 6 in the position 6 p25.2. The seven exons span 19.8 kb and the gene (NCBI ID:4835) can be transcribed in four different transcripts (Figure 1A), but the longest three transcripts encode the commonly known protein of 231 amino acids (aa) with a molecular weight of 25,956 Da. The *NQO2* gene locus is highly polymorphic and contains almost 9000 allelic variants, which is over the average of a typical gene of this length. By comparison, its paralog *NQO1*, which codifies for a longer protein of 274 aa contains around 7000 allelic variants. Several cis-elements can be identified within the *NQO2* gene promoter, such as SP1 binding sites, CCAAT box, xenobiotic response element (XRE) and an antioxidant response element (ARE) (Figure 1B).

The transcription factors binding to these elements must modulate tissue-specific expression of the *NQO2* gene and participate in the response to xenobiotics and antioxidants.

Most *NQO2* polymorphisms are found in introns (7013) and in the non-coding regulatory and promoter regions at 5′-end up to 2 kb upstream exon 1 (828) and in the 5′-UTR of the transcripts (428), including several insertion/deletion (I/D) polymorphisms. A total of 302 SNPs and 17 frameshift I/D are found in exons. The exon SNPs may impact the catalytic activity, independently of the expression levels, while other polymorphisms may influence or fully prevent the expression of the full-length protein. Unfortunately, as shown in Figure 1B, only a small portion of *NQO2* polymorphisms were characterized from the functional point of view. In addition, there is a substantial confusion with the nomenclature of the studied *NQO2* polymorphisms, because many studies were published before an unequivocal SNP identification system was introduced. In fact, scientists used as a reference for SNP location different gene/transcript sequences, which were subjected to frequent updates, leading to three or even five alternative names for the same SNP cited in the literature. To overcome this problem, we searched for rs-based codes in the NCBI SNP database (dbSNP) to identify all ambiguous SNPs cited in the literature. The rs codes and alternative SNP names are reported in Figure 1B and in Table 1.

The most studied *NQO2* gene variant is a 29-base pair (bp)-insertion (I-29) or -deletion (D-29) located in the gene promoter. The I-29 sequence creates a recognition site for the transcriptional repressor Sp3 [16], and thus, the presence of I-29 in the promoter leads to a decreased *NQO2* expression. In fact, the I-29 promoter compared to the D-29 promoter or to a promoter containing an alternative 16 bp insertion sequence (I-16) demonstrated significantly lower *NQO2* expression and lower enzyme activity [16,17]. An independent study compared *NQO2* mRNA levels in breast cancer tissue with D-29 or I-29 homozygosity and confirmed lower expression for the I-29 variant [18]. A similar effect on NQO2 expression was described for rs2071002 (+237 > C, also known as −102A > C) SNP located in the 5′ untranslated region (UTR) of *NQO2* gene. In fact, the C variant showed significantly higher *NQO2* gene expression compared to its A-containing counterpart, which was attributed to the increased Sp1 binding [18]. It is not known if other SNPs in the 5′-UTR and promoter regions influence NQO2 expression at mRNA or protein level, but some SNPs were associated with lower NQO2 activity. In fact, the 3423 G (rs2070999) and 3777 G (rs2071001) alleles were reported to have reduced activity in bladder, ovarian and prostate tumor samples [19,20]. Considering that these SNPs are located in 5′-UTR outside the coding region, they cannot directly influence the activity, but they likely modulate NQO2 expression at the RNA or protein translation level. The direct effect on enzymatic activity may have only the SNPs located in exons by introducing aa substitutions. Nevertheless, only a few studies have been carried out to verify the sequence-activity relationship for some *NQO2* exon SNPs. For example, the exon 3, T14055C (rs1143684), C allele was associated with a lower relative NQO2 activity in human ovarian and bladder samples [19]. In fact, the C variant, coding for NQO2-L47, with leucine at position 47 showed significantly reduced activity compared to the T NQO2-F47 wild-type variant with phenylalanine at position 47. In addition, L47 was less stable towards proteolytic digestion and thermal denaturation than the F47 wild-type variant. Both forms also showed some differences in the kinetics of resveratrol inhibition [21]. Two other promoter polymorphisms, C3395G (rs2070998) and I/D-29, and one exon 1 SNP A3968C (rs2071002), were analyzed for a functional role, but no differences in NQO2 activity or expression were identified in bladder and ovarian tumors [19], in contrast to later studies discussed above, suggesting that the genetic variant effect on the *NQO2* expression might be tissue-dependent [17,18].

Recent genetic studies have revealed that many functionally defined and some undefined *NQO2* polymorphisms can alter cancer susceptibility and progression, modify the response to chemotherapy, but also may predispose to Parkinson’s disease and certain neurological dysfunctions. The results of these studies are described in the following chapters addressing the role of NQO2 in disease.

### 1.2. The Role of NQO2 in Drug Metabolism

Drug metabolism consists of a series of enzymatic steps that encompass all the necessary reactions to eliminate molecules from living organisms. Mainly situated in the liver and the kidneys, they nevertheless also exist in other organs, such as in the derma, the gut and the brain, among others. Drug metabolism is traditionally divided into two phases: functionalization (Phase I) and conjugation (Phase II). Among the functionalization steps, the cytochrome P450 family of enzymes is the key component of the whole process, as these enzymes can introduce in a molecule—whether of endobiotic or xenobiotic origin—a hydroxyl moiety that will be recognized by the subsequent set of conjugating enzymes of Phase II. Phase II comprises enzymes that will conjugate the molecule with a highly soluble component, favoring the elimination by the urine or the feces. These steps have been wonderfully summarized by Kramer and Testa in a series of key reviews [22,23,24]. Besides those straightforward steps, there is a series of enzyme-catalyzed processes that are less easy to categorize in these two phases.

**Figure 2:**
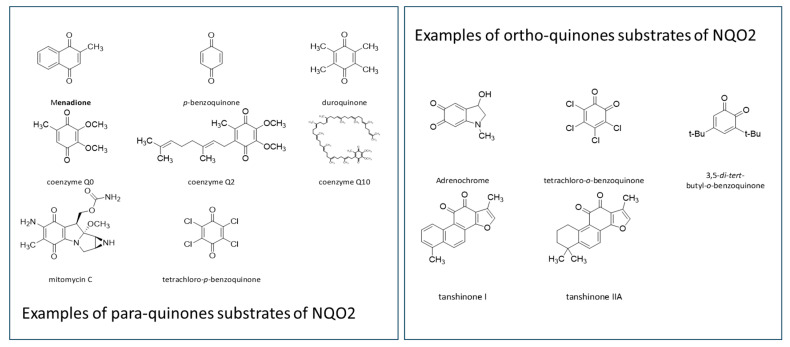
Chemical structures of NQO2 substrates classified as para- and orthoquinones.

Quinones (Figure 2) are often highly toxic molecules, the detoxication of which might depend on different enzymes. If non-aromatic quinones such as camphoroquinone can be reduced to diols by the surprising 3-hydroxysteroid-dehydrogenase [25], aromatic quinones are reduced by either the major protein NQO1 [26,27] or by the less universal one, NQO2 [7]. Quinones can cycle between quinones and unstable diols in the presence of oxygen, leading, during those cycles, to the production of reactive oxygen species as by-products which are extremely harmful to cells [28]. So, a special system has been developed to eliminate these molecules by reduction to diols, and then by conjugation with hydrosoluble moieties that favor their elimination from the body: the quinone reductases.

Based on the origin of these molecules, we can distinguish (i) endobiotic quinones, such as those derived from steroids (estrone-quinone) [29], coenzymes Q or ubiquinones, (ii) naturally occurring quinones from plants or insects (such as dunnione) [30] among many others, or (iii) synthetic quinones from pollutants or drugs as a recent review presents some of the drugs concerned by this pathway [2]. Besides pure para- or ortho-quinones, semiquinones, imine-quinones, other molecules can be reduced by NQO1 and 2 (Figure 2). Briefly, NQO1 as indicated in its name uses NAD(P)H as a co-substrate. This substance being in the mM range concentration in the liver [31], it is, most likely, the main reductase of quinones, in almost every tissue, with special mention of the liver and kidneys, as they are the main organs responsible for drug metabolism. To the contrary, NQO2 does not recognize NADH as a co-substrate, but only its precursor, namely NRH and its derivatives and analogues, N-benzyl- or N-methyl-dihydronicotinamide [32]. This surprising and often mis-regarded specificity renders the enzyme “mysterious” and as a consequence much less studied [1]. Indeed, the very existence of this metabolite has been poorly explored for many years and only more recently it has drawn attention as an important precursor in the synthesis of NAD(+) [3,33,34], while its unreduced form NR has been identified in dietary sources and defined as a new anti-aging vitamin [35,36]. This led some to hypothesize that NQO2 might have lost its catalytic capacity during evolution [4]. To rule out this possibility, one has to recall that NQO2 activity can be recorded in the presence of added NRH and towards a large series of quinone substrates [10,37]. More importantly, expressing both NQO2 and the main Phase II enzyme, the conjugating UDP-glucuronosyltransferase (UGT) in SH-SY5Y cells, it was possible to demonstrate the appearance of menadione glucuronides if NQO2 was expressed and incubated in the presence of its co-substrate, while in the absence of NRH, menadione glucuronide remained undetectable, strongly suggesting that the reduction of menadione to its diol was enough to permit UGT to conjugate it [9]. We also hypothesized such a role in the UGT/NQO2 activity balance [38]. Further studies are necessary to decipher this equilibrium. Furthermore, there is no compendium of NQO2-specific substrates, although CB1954 (tretazicar^®^) and dunnione have been reported to be exclusive [1] and marginally specific NQO2 substrates [30], respectively.

A large panel of compounds, especially drugs and pharmacological agents were reviewed as suspected substrates of NQO2 [2]. Nevertheless, the actual role of NQO2 in this reduction context is hard to precisely pinpoint, because most of the time, the studies are performed on whole organism(s) where the respective roles of NQO1 and 2 are hard to distinguish.

The toxifying function of NQO2, anticipated in the introduction, was first described in the case of menadione [39], then for a series of substrates and other unrelated compounds [11,13,40,41]. For example, it was reported that NQO2 mediates the generation of ROS by acetaminophen, and thus its toxicological side-effects [42], leading to a new concept that NQO2, despite its reductase capacity, might have been under special circumstances an enzyme that indirectly leads to the enhancement of ROS production. Indeed, unstable diols, generated during the NQO2-mediated redox reactions, may recycle back into the original quinone in the presence of oxygen, leading to a futile cycle during which massive amounts of ROS are produced [43], a feature reported occasionally for NQO1 [44].

Such capacity was pinpointed to acetaminophen, and could also be questioned for dopamine-quinone, as both those molecules have been co-crystallized with NQO2 [45] or docked in the binding site of the enzyme [42].

Those molecules, through their reductions by NQO2, may very well function as a novel mechanism generating (dopamine-quinone) [43] or augmenting (acetaminophen) [46] their off-target toxicities. NQO2 catalyzed the reduction of quinone-like metabolites derived from other drugs such as clozapine, 4′-hydroxydiclofenac, mefenamic acid, amodiaquine, and carbamazepine. Its detoxifying or toxifying function in these oxidation/reduction reactions requires many more bench experiments [47].

In conclusion, NQO2 is part of the Phase I drug metabolism, rather marginally investigated with respect to other Phase I enzymes because of the unknown bioavailability of its co-substrate, NRH. With regard to the NADH concentration—the NQO1 co-substrate—NQO2 might have a minor detoxification role. Nevertheless, recent studies seem to point out a possible role of NQO2 in the generation of a futile cycle, according to which ortho-quinone reduction by NQO2 leads to unstable aromatic diols that could become a major source of ROS production in the tissues, where it is more expressed. According to this, NQO2 might be an important player in both cancer and neurodegenerative diseases.

## 2. The Functional Role of NQO2 Polymorphic Variants in Pathology

### 2.1. Cancer

#### 2.1.1. Breast Cancer (BC)

A possible role of *NQO2* in tumorigenesis was postulated based on several genetic studies associating the risk of BC with certain *NQO2* polymorphisms.

The most widely studied *NQO2* polymorphisms are the 29-base pair (bp)-insertion/deletion (I-29/D-29) located in the *NQO2* gene promoter, influencing NQO2 expression and activity [16] and C allele of rs2071002 (−102 A > C) SNP in 5′-UTR causing higher NQO2 expression (Table 1).

**Table 1:** Polymorphic variants of *NQO2* associated with human diseases or drug adverse effects. * The following alternative names for each SNP are indicated: rs code (NCBI dbSNP system), 1 to 4 different alternative names as reported in the literature and the genomic position on chromosome 6 (NC_000006. 12). For further information, see Supplementary Table S1.

Polymorphism Names * and Location	Allele	Possible Molecular Function	Ref.	Pathology	Higher Risk Allele	Clinical Reference
29 bp-I/D Promoter	D	No Sp3 site Higher *NQO2* expression and activity	[16,17,18,48]	Breast cancer	I	[18]
				Parkinson’s Disease Idiopathic	D	[49]
				Schizophrenia	D	[50]
				Methamphetamine-related psychosis	D	[51]
				Alcoholism and alcohol withdrawal symptoms	D	[52]
	I	Sp3 site present Lower *NQO2* expression and activity		Early breast cancer	I	[53]
				Chronic Alcoholic pancreatitis	No	[54]
				Primary Breast cancer	I	[48]
				Parkinson’s Disease Idiopathic	No	[55]
				Papillary thyroid microcarcinoma (PTMC)	I	[56]
rs2070999 3423 G>A Promoter 2 kb upstream var. 2999495	A	Lower *NQO2* activity	[19,57]	Esophageal cancer (EC)	A	[57]
				Esophageal cancer (EC)	No	[58]
	G	Higher *NQO2* activity		Gastric cancer	No	[59]
				Bladder cancer	A	[19]
				Prostate cancer	No	[20]
rs20710001 3777 A>G 2999878	A	Higher NQO2 activity	[2,19,21]	Bladder cancer	G	[19]
	G	Lower NQO2 activity		Ovarian cancer		
rs2071002 +237 A>C −102 A>C 1507 C>A 5′-UTR variant 3000069	A	Abolishes Sp1 site Lower *NQO2* expression	[18,48]	Breast cancer	A	[18]
				Primary breast cancer	C	[48]
	C	Sp1 site present Higher *NQO2* expression		Sporadic breast cancer	C	[60]
				Epithelial ovarian cancer	A	[61]
rs2071003 1536 C>T Intron 1 variant 3000098	T	No MZF-1 binding site Low *NQO2* expression	[62]	Clozapine-induced Agranulocytosis	T	[62]
	C	MZF-1 binding site High *NQO2* expression				
rs2071004 1541 G>A Intron 1 variant 3000103	A	Low *NQO2* mRNA	[63]	Agranulocytosis	A	[63]
	G	High *NQO2* mRNA				
rs17136117 86 A>G Exon 3 Missense (Glu>Gly) variant 3010156	A	Unknown		Sporadic Breast cancer	G	[60]
	G					
rs1143684 139 C>T 14055 T>C 372 T>C Exon 3 Missense (Phe>Leu) variant 3010103	T (Phe)	Higher NQO2 activity	[19,21,48,62]	Primary Breast cancer	T	[48]
				Breast cancer	No	[64]
				Localized Prostate cancer	C	[65]
	C (Leu)	Lower NQO2 activity		Pancreatic cancer	No	[66]
				Bladder cancer	No	[67]
				Bladder cancer	C	[19]
				Ovarian cancer	C	[19]
				Cognitive decline	C	[68]
				Clozapine induced- Agranulocytosis	C	[62]
rs1049115 202 G>A Pro110Pro silent Exon 5 3015556	A	Silent mutation	[62]	Clozapine induced- Agranulocytosis	A	[62]
	G					
rs199706530 418−2 A>G 3′-end of intron 5 3016882	A	Normal splicing	[69]	Hereditary Breast and Ovarian Cancer	G	[69]
	G	Aberrant splicing: no acceptor splice site, truncated transcripts *w*/*o* exons 5 and 6				
rs10223369 C>T Intron 6 variant 3017940	T	Unknown		Localized Prostate cancer	T	[65]
	C					
rs6920900 G>C 3′-UTR variant 3020755	C	Unknown		Localized Prostate cancer	C	[65]
	G					
rs927340 G>A 3′-UTR 3023484	A	Unknown		Ovarian cancer	No	[70]
	G					

An important Chinese study involving more than a thousand BC patients and cancer-free controls clearly indicated a high confidence of decreased risk of BC correlating with “gain of function” D-allele (odds ratio (OR), 0.76; *p* = 0.0027) and C allele of rs2071002, confirming the correlation between lower NQO2 expression and higher breast cancer susceptibility [18]. D-allele, associated with high NQO2 expression was particularly rare in estrogen receptor-positive breast carcinomas with wild-type p53, thus supporting a hypothetical functional interaction between NQO2 and p53 [18]. *p53* is a highly penetrant breast cancer susceptibility gene and one of the most important breast tumor suppressors. The loss of both *p53* and *BRCA1* causes rapid formation of mammary carcinomas [71,72]. The crosstalk between *NQO2* and *p53* implies a potential modulating effect of *NQO2* on breast cancer as a negative modifier of carcinogenesis. This hypothesis is in accordance with the observation that NQO2 catalyzes the reduction of electrophilic estrogen quinones, carcinogens in mammary glands, and thereby acts as a detoxification enzyme [73]. In fact, Gaikwad et al. successfully demonstrated that estrogen-3,4-quinone is metabolized by NQO2 and proposed that estrogen quinones are endogenous biological substrates of NQO2 and NQO1 [29], as NQO2 seems to be more efficient in reducing ortho-quinones than its homologue [37].

The findings of Yu et al. [48] have not been confirmed yet in non-Chinese populations, and most subsequent studies were confined to BC patients and dealt with other SNPs or with a prognosis of cancer progression and patients response to chemotherapy [18,48,53,60,74,75], as listed in Table 1. For example, in another case–control study, the same tri-allelic polymorphism, genotyped in 1164 BC patients and compared to 1701 cancer-free controls, was associated with BC risk, especially for the luminal-like subtype but not with HER2-positive or triple-negative subtypes [76]. *NQO2* was studied through exon sequencincing as a non-canonical risk factor candidate for Hereditary Breast Ovarian Cancer (HBOC) susceptibility, beyond *BRCA1-2* genes [77], in a sample of 200 individuals with HBOC screening [69]. rs199706530 *NQO2* (418-2 A>G), a splice acceptor site variant generating two aberrant transcripts, was identified in a patient and classified as pathogenic or likely pathogenic variant [69]. In another study, rs1143684 (Phe > Leu) was correlated to a worse prognosis, according to the hormonal receptor status in ER/PR negative patients, although Choi et al. reported a negative association of rs1143684 SNP with BC progression-free survival [64].

#### 2.1.2. Other Cancers

Several lines of evidence indicate that *NQO2* may play a role in other cancers, such as colorectal, ovarian, prostate, gastric, pancreatic, bladder and thyroid cancers as supported by genetic association studies listed in Table 1, or by a few functional studies. The pathogenesis and development of colorectal cancer (CRC) is a multi-step process, and the majority of CRC cases (around 70%) are caused by chromosome instability (CIN). Oncomine, a web-based microarray gene expression data-mining platform analysis showed that NQO2 mRNA is overexpressed in CRC characterized by CIN, particularly in cells showing a positive KRAS (Kirsten rat sarcoma viral oncogene homolog) mutation [78]. In another study, the authors have performed a genome-wide analysis of *Long non-coding RNAs* (lncRNAs) expression to identify novel targets implicated in CRC progression leading to liver metastasis. The primary function of lncRNAs is the epigenetic regulation of protein-coding genes [79]. The authors identified significant downregulation of NQO2 mRNA levels and six strongly NQO2-associated lncRNAs in CRC liver metastasis foci [80]. Thus, *NQO2* might play a positive role in the establishment and maintenance of CIN, but a negative role in the invasive behavior at final stages of CRC progression. No *NQO2* polymorphisms have been associated so far with the susceptibility to CRC.

The role of *NQO2* in prostate cancer (CaP) is also dual. On one hand, a presumed gain-of-function allele of rs1143684 polymorphism in exon 3 has been associated with a higher risk of progression of prostate disease (HR = 1.51; *p* = 0.03) and with shorter time to biochemical recurrence of advanced CaP, in a study involving a total of over 700 patients [65]. In particular, 526 men with localized disease were genotyped for seventy-one SNPs in genes related to estradiol metabolic pathways, and subsequently sixteen SNPs were validated in 213 men with locally advanced prostate cancer. Relatively to *NQO2*, only in the first group of patients were three SNPs (rs10223369, rs1143684 and rs6920900) found to be significantly correlated with time to biochemical recurrence (BCR) and with a higher risk of progression. These findings suggest a potential role of the *NQO2* pathway in the early stage of disease during which other estrogen-related genes are involved in the mechanisms associated with cancer progression. Accordingly, NQO2 is highly or moderately expressed in human prostate tumors, where it may mediate the inhibitory effects of resveratrol in CWR22Rv1 CaP cells, by blocking the oncogenic action of AKT [81,82], but may be involved in the progression and metastatic behavior of other CaP cell lines [83].

Different *NQO2* polymorphisms have been implicated in a few other neoplastic diseases according to Table 1. However, only three studies confirm an association with functional polymorphisms of *NQO2*, suggesting both a positive as well a negative role of *NQO2* in carcinogenesis. For example, the 5′-UTR frameshift variant I-29, causing lower expression and activity of NQO2 has been associated with aggressive clinical phenotypes of papillary thyroid microcarcinoma (PTMC) [56,84], while a high NQO2 expression allele A of another 5′-UTR polymorphism rs2071002 (−102 A>C) has been associated with more aggressive phenotype and reduced survival of patients with Epithelial ovarian carcinoma [61]. NQO2-3423 G>A (rs2070999) causing higher NQO2 activity in the ovarian tumors [19] was implicated in predisposition to bladder and ovarian cancers, suggesting that NQO2 might be a therapeutic target in these types of cancer [19,85]. However, other SNPs like rs927340 and rs1143684 C>T, were not associated with a higher risk of ovarian [70] and bladder cancer [67], respectively. Thus the question if hypermorphic NQO2 genetic variants promote bladder and ovarian carcinogenesis, remains open.

In conclusion, several genetic studies suggest rather an important role of *NQO2* in breast carcinogenesis, where it seems to be a tumor suppressor at early phases and tumor promoter at later stages of breast cancer development, but there are no functional studies in support of this hypothesis. The tumor-promoting function emerges as a prevalent role of NQO2 in prostate and colon cancer, where few functional studies seem to confirm this hypothesis, but other studies are in line with the opposite hypothesis. The role of *NQO2* in other tumor types seems to depend strongly on other genetic and environmental factors, but further studies are needed to decipher the actual role of *NQO2* in cancer.

### 2.2. Neurodegeneration 

The oxidative stress imbalance and the increased production of ROS, mainly superoxide radicals, are involved in the etiopathogenesis of neurodegenerative diseases. Due to the known role of NQO2 in the regulation of redox balance, this enzyme could have a potential role in the etiology of neurodegenerative diseases including Alzheimer’s disease (AD) [86] and PD [12].

In 2001, a population-based case–control Japanese study highlighted NQO2 as a possible target in PD identifying a positive association of a common, non-familiar form of PD with a “gain of function” genetic variant in the *NQO2* promoter region, without Sp3 transcriptional suppressor binding site (see Table 1). The frequency of this polymorphism was 3.46 times higher in PD patients than in healthy subjects [49]. This correlation was confirmed in a case–control study that further documented the presence of three variants of *NQO2* promoter (I-29, I-16 and D alleles): subjects carrying the D allele showed an increased susceptibility to PD [17]. Higher *NQO2* gene expression was, however, associated only with the promoter containing the D and/or I-16 allele, and consequently, the higher correlation between PD risk and the D allele was hypothesized to be caused by higher NQO2 activity and increased levels of ROS in the presence of dopamine. Nevertheless, these studies were in contrast with another study in which no correlation with PD was detected for any NQO2 allele combinations (I/D, 29 base pairs) in a population-based case–control study (190 idiopathic PD cases vs. 305 unrelated matched controls), suggesting that the intrinsic characteristics of a studied population can influence the PD-driving effect of D variants [55].

The possible role of *NQO2* in PD is confirmed by many other studies. The oxidative metabolism of dopamine with excessive ROS production in *substantia nigra* is considered to be responsible for dopaminergic neurodegeneration found in PD brains. The higher expression of NQO2 was demonstrated to exacerbate dopamine quinone toxicity in several cell systems. In support of the toxifying role of NQO2 in dopamine metabolism, the overexpression of NQO2 in Chinese hamster ovary (CHO) cells exposed to exogenous ortho-catechol-quinones (e.g., dopachrome, aminochrome and adrenochrome) was responsible for increment the production of ROS [37], equally to what was demonstrated in human leukemic cells (K562 cells) and neuroblastoma (SHSY5Y) cells [43]. The conclusions of these data suggested the role of NOQ2 in the early stages of the neurodegeneration process. Moreover, NQO2 may mediate the toxic effects of Parkinsonian toxins, such as paraquat (PQ) in astroglial cells [11] during the induction of PD. In fact, it was shown that the specific inhibition of NQO2 by NMDPEF (also known as S29434) can block PQ toxicity as demonstrated in a simple model of PD-like seizures in rats exposed to PQ infused into the *substantia nigra*. In this experimental model, the protection due to NQO2 inhibition was associated with a potent reduction in oxidative stress in astrocyte cultures and brain specimens, suggesting a pro-oxidative function of NQO2 in PQ redox cycling [11].

Further studies have associated higher NQO2 expression levels with autophagy dysfunction and poor neuroprotection in astrocytes exposed to Parkinsonian toxins such as PQ and 6OHDA [12,13]. Astrocytes play an important role in dopamine metabolism and their dysfunction contributes to *substantia nigra* degeneration [87,88]. Importantly, the analysis of Gene Expression Omnibus datasets showed elevated *NQO2* gene expression in the blood cells of early-stage PD patients [13], in line with the proposed toxifying function of NQO2 in nigrostriatal degeneration [43].

Some evidence has also been accumulated about the potential role of NQO2 in AD pathogenesis. NQO2 protein levels were found higher in the hippocampus and brain cortex post-mortem samples of AD patients [89,90]. More recent data suggest that a higher expression and activity of NQO2 contributes to memory impairment in mouse models of AD (5XFAD), while novel NQO2 inhibitors improve cognition and reduce pathology in the brains of the experimental mice [91]. These data confirm the previously formulated hypothesis that QR2 is a removable memory constraint in rodents [92], The mechanism behind this effect is unclear but might be related to *NQO2*-dependent gene expression changes. In fact, the deletion of *NQO2* gene by CRISPR-Cas9 technology in HCT116 cancer cell line caused remarkably opposed changes in energy metabolism gene expression patterns compared to AD brains [93] associated with microglia and astrocyte activation [91]. Importantly, the attempts to inhibit NQO2 by potent inhibitors led to different levels of protection from insults in PD or AD models [14,91].

So far the genetic studies have not identified *NQO2* gene variants predominantly expressed in AD patients or related to the pharmacogenomics of AD therapy, although one study reported the involvement of an exon 3 missense variant of *NQO2* in cognitive decline [68]. Future studies should fill in this gap.

### 2.3. Memory and Brain Pathophysiology

There is an association between high ROS levels and age-associated impairment in learning and memory as well as Alzheimer’s disease [94]. Thus, the proteins involved in oxidative stress regulation, such as NOQ2 may play a role in cognitive behaviors. R. Quirion’s group addressed molecular differences and alterations in hippocampal gene expression, involved in long-term memory formation, in aged rats and found NQO2 upregulation correlated with memory deficits. In addition, NQO2 was found overexpressed in the scopolamine-treated rats [95], which is another psycho-pharmacological model of learning impairments. Importantly, the selective NQO2 inhibition by S26695 or by S29434 (8 mg/kg), chronically injected intracerebroventricularly, significantly reversed scopolamine-induced amnesia, evaluated after various behavioral tasks, while adult NQO2 knock-out mice (NQO2^−/−^) showed facilitated learning abilities in learning tasks without alterations in behaviors related to anxiety, depression, and psychosis [89]. Similarly, it was demonstrated that NQO2 was negatively involved in memory acquisition mediated by muscarinic acetylcholine receptors (mAChR) [96,97].

Few genetic association studies indicate a potential function of NQO2 in brain pathophysiology, and most of them support a negative role of *NQO2* (Table 1). The 29 bp insertion/deletion (I/D) polymorphism in the promoter region of *NQO2* was found to be involved in the pathogenesis of alcoholism and alcohol withdrawal syndrome [52]. D variant of the *NQO2* gene was significantly more frequent in alcoholic patients with delirium tremens and with hallucination than in controls, suggesting that high expression of NQO2 may facilitate alcohol dependence. This gain-of-function D allele was positively associated with psychiatric disorders, suggesting that a higher NQO2 expression might be associated with susceptibility to some forms of schizophrenia [50] and methamphetamine-associated psychosis [51]. Another study on a cohort of 722 older individuals showed that the rs1143684 *NQO2* (NQO2-L47) was significantly associated with delayed memory recall [68] without any decline in other cognitive abilities. This polymorphic form of *NQO2* is presumably less stable [21], which would be in contrast to the body of evidence suggesting a positive correlation between memory impairment and increased NQO2 expression, as discussed above [89,90,95].

## 3. NQO2 and Pharmacogenomics

Pharmacogenomics (PGx) studies identify genetic factors influencing both drug pharmacokinetics and pharmacodynamics [97], mainly focusing on polymorphic variants in adsorption, distribution, metabolism and excretion (ADME) genes [98]. The most common genetic alterations studied by PGx are represented by genomic insertions and deletions, genetic copy number variations (CNVs) and single-nucleotide polymorphisms (SNPs). SNPs are single-nucleotide differences in the DNA sequence, and when occurring within a gene coding sequence or in a regulatory region, they may play a functional role.

The high-throughput PGX genotyping approaches, from targeted to genome-wide association studies (GWASs) have allowed the implementation of PGX findings for the discovery of biomarkers associated with the individual risk of adverse drug reactions and drug efficacy [99]. To date, the US Food and Drug Administration (FDA) has recognized more than 250 biomarkers in CYP450 [100], transporters and other drug metabolism-relevant genes, and based on these findings provided recommendations for therapeutic decision-making (https://www.fda.gov, accessed on 12 October 2023). In this scenario, *NQO2*, a phase I drug metabolism gene involved in the bioactivation of antitumoral drugs to reactive hydroquinones, is a valid candidate biomarker that merits more attention.

### 3.1. Effects of NQO2 Polymorphisms on Cancer Therapy

Among numerous polymorphisms present in the *NQO2* gene locus, only few polymorphic variants have been associated with a functional role in the response to therapy, drug toxicity and/or metabolism.

Jamieson et al. demonstrated that *NQO2* rs1143684, missense Phe > Leu SNP, related to lower enzyme activity, modulates the adjuvant doxorubicin and cyclophosphamide (AC) efficacy and tamoxifen toxicity in 227 early breast cancer (BC) patients, with estrogen receptor (ER)- and progesterone receptor (PR)-negative disease [53]. This SNP was subsequently proposed as a potential BC prognosis biomarker correlated to the disease stage and PR expression status [74,101]. In contrast, the same authors found no correlation with overall survival, progression-free survival, or toxicity for the triallelic *NQO2* promoter variant I-29, I-16, and D alleles in 223 BC patients. This is surprising since previous studies showed the association between the incidence of BC or PTMC aggressiveness and 29 bp-I/D polymorphism, where 29 bp-I was a risk allele [18,56] and a putative prognostic marker for PTMC [56]. In another study, individuals carrying at least one minor allele of rs1143684, missense Phe > Leu SNP, causing lower NQO2 activity, were found to be slow metabolizers of epirubicinol/epirubicin, with higher exposure to the toxicity of these drugs [102].

### 3.2. Effects of NQO2 Polymorphisms on Other Drug Metabolism

NQO2 was also studied for its ability to catalyze the reduction of quinone-like metabolites derived from different drugs, including acetaminophen, clozapine, diclofenac, mefenamic acid, amodiaquine and carbamazepine [47,103]. NQO2 appeared less active or inactive towards most of the chemically reactive drug metabolites, including 5-hydroxy diclofenac-derived quinone-imine and other quinones when compared to NQO1. Thus, it is not clear what the in vivo role of the NQO2-catalyzed reduction of quinone-like metabolites is, although hepatic expression levels of NQO2 are higher and less variable compared to NQO1. However, for the clozapine nitrenium ion reduction, NQO2 activity, but no NQO1 activity, was observed. During clozapine metabolism, an atypical antipsychotic drug, the lower expression of *NQO2* 1541 G > A, was reported as correlated to a higher risk for clozapine-induced agranulocytosis (CIA) in 310 Dutch psychiatric patients treated with clozapine compared with control subjects [63]. In this population, 31 patients developing agranulocytosis were homozygous wild type or mutant for this variant. The same conclusion was achieved by Ostrousky et al. in an Israeli study with 98 clozapine schizophrenic patients where 18 of them, all heterozygous for the same variant, developed CIA [62]. It was reported that *NQO2* 1541 G>A mutation disrupts the Zinc Finger Transcription Factor MZF1 binding site, which is expressed in myeloid cells and involved in granulopoiesis [104]. Instead, a lower expression of NQO2 mRNA was demonstrated in neutrophils of CIA patients compared to controls. The other SNPs detected in this study and significantly associated with CIA were the 1536 C>T, the 372 T>C Exon 3 and the silent variant 202 G>A exon 5. In all 18 patients, the first two intronic variants were also heterozygosity [62]. Relating to acetaminophen (paracetamol) toxicity, Miettinen et al. demonstrated in vitro that NQO2 might be considered as an off-target for acetaminophen-mediating superoxide production and modulation of Ca2+ levels in cultured HeLa cells [42]. Acetaminophen represents a weak NQO2 substrate, but the mechanism underlying the in vivo involvement of NQO2 in acetaminophen metabolism is still debated and the NQO2 cosubstrates might be responsible for the interindividual variability to acetaminophen treatment. The liver and kidney are the principal sites of acetaminophen toxicity and also the sites where *NQO2* is highly expressed. So, NQO2 can modulate acetaminophen-associated production of ROS, particularly superoxide anions, in humans as well as in cultured HeLa cells which induces the modulation of Ca2+ levels. This suggests its potential role as a novel mechanism correlated to acetaminophen toxicity [42]. Moreover, the Nqo2 29 bp-I/D polymorphisms were correlated to the etiology of prolonged type methamphetamine (MAP)-related psychosis in 191 Japanese patients with MAP dependence and psychotic disorders compared to 207 matched normal controls without past and family history of drug dependence or psychotic disorders [51]. The study demonstrated prolonged-type MAP psychosis in 11.7% of *NQO2* D/D patients than in 4.8% of controls and that a lower expression of *NQO2* may be associated with this condition.

## 4. Conclusions and Future Perspectives

**Figure 3:**
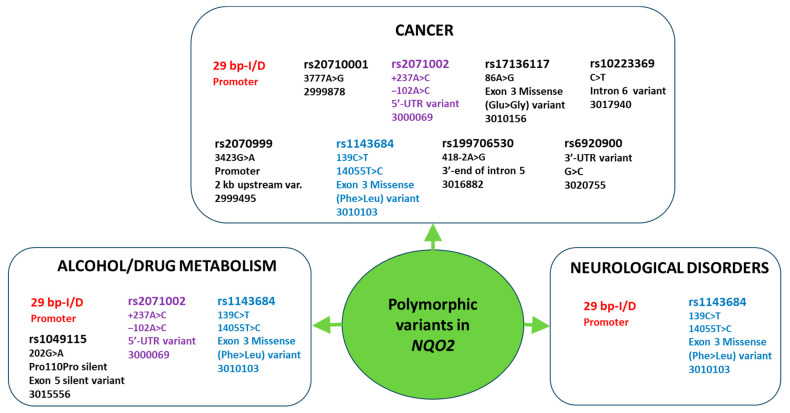
All identified polymorphic variants in *NQO2* associated with human disease.

Our review depicts *NQO2* as a highly polymorphic gene, implicated in several forms of cancer, neurodegenerative diseases and other pathologies as well as in drug-adverse effects (Figure 3). To our knowledge it is the first systematic review focusing on *NQO2* polymorphisms and PGx.

Genetic studies suggest rather an important role of *NQO2* in breast carcinogenesis, but there is no functional research to mechanistically explain these findings, since the papers regarding the role of NQO2 in p53 stability have been retracted. The tumor-promoting function emerges as a prevalent role of *NQO2* in prostate and colon cancer, where few functional studies seem to confirm this hypothesis, but there are also studies in line with the opposite hypothesis. Thus, further research is needed to decipher the actual role of *NQO2* in cancer.

The evidence from cell and animal models suggests that NQO2 is implicated in AD and high NQO2 protein levels are found in some AD brains, but the genetic evidence is missing. Several studies also support a toxifying role of NQO2 in the early stages of PD and these findings correlate with the high expression of NQO2 in blood of early PD patients, but genetic evidence supporting the involvement of “gain-of-function” polymorphic variants in PD is conflicting. This clearly indicates that other studies are required to support the role of *NQO2* in neurodegenerative disorders.

In addition, our understanding of the *NQO2* functions in normal physiology and disease is fragmentary, and further research is needed to fill numerous gaps. The same is true with respect to PGX studies of polymorphic variants in the *NQO2* gene, where relatively little evidence is reported in the literature. This is in strong contrast to the emerging evidence suggesting that plenty of known drugs interact with NQO2. In fact, the literature is full of examples of drugs and compounds that have been found to work as NQO2 inhibitors including imatinib and other ABL kinase inhibitors [105], imiquimod [106], chloroquine and hydroxychloroquine [107], imidazole derivatives and other cancer drug candidates [108,109,110,111]. Many drugs or their quinone metabolites are known NQO2 substrates such as mitomycin, tretazicar and other anti-cancer drugs [40], paracetamol, clozapine, carbamazepine and others [47]. NQO2 is also inhibited by natural polyphenols with anti-cancer and anti-inflammatory properties like curcumol, apigenin and luteolin [5,112]. Many other drugs are expected to be targets of NQO2 since the enzyme occupies the absolute first position among the most frequently identified secondary drug targets in bioinformatic studies [15]. Thus, the very existence of many potent NQO2 inhibitors—if their specificities are confirmed by direct studies, particularly in humans—would also open up a large field of investigation on the use of these inhibitors to limit the metabolism of relevant drugs and the activity of which might be limited by this particular NQO2-driven pathway. In order to decipher this complex picture, many further studies will be needed shortly.

The obvious limitation of the present review is a very small number of polymorphic variants described in the literature and discussed here (less than 20) with respect to the total number of identified polymorphisms in the *NQO2* gene. This number, according to *NCBI SNP gene viewer*, reaches almost 9000. In particular, the available literature does not cover any of 17 frameshift variants and 13 variants coding for truncated proteins, due to stop codon insertions. There is only one case of a splice acceptor variant, out of 14 possible, which is poorly characterized in the source study [69]. As such, genetic alterations might have a dramatic, or just stronger impact on NQO2 function than most SNPs. Finally, *NQO2* contains 193 missense variants, but only two have been characterized by the available reports. This overview clearly shows that our understanding of *NQO2* genetic variants is just a drop in the ocean compared to what can be achieved in the future.

Another limitation of this review is the relatively small number of PGx studies addressing NQO2 role in drug metabolism. Very often, only one report is available, and there are no studies to verify its findings. The limited attention to NQO2 as a drug target and drug metabolizer is most likely dependent on the poor understanding of the regulatory mechanisms of NQO2 activity and the availability of its main co-substrate NRH. The same is true with respect to the NQO2 role in physiology and pathology. In fact, closely-related NQO1, that uses NADH, a typical co-substrate for oxidoreduction reaction, is at least ten times more studied with regard to polymorphic variants than NQO2. Recent systemic reviews confirm that NQO1 PGx is much more advanced compared to NQO2 PGx and addresses specific topics [113,114,115]. We can expect that a better grasp of the regulatory mechanisms of NQO2 enzymatic activity would trigger a stronger interest and faster progress in our understanding of NQO2 genetics, biology and pharmacology in the future. Nevertheless, it should be emphasized that unlike other drug metabolism genes with an established match between genetic variants and metabolizer phenotypes, NQO2 polymorphisms are relatively well-associated with disease risk and progression. With the expected progress of PGx studies and identification of new drugs dependent on NQO2 genetics, the information about an obvious link to disease risk should be carefully stored and shared to guide the decision making with regard to drug choice and dosing.

In conclusion, *NQO2* represents undoubtedly a new frontier of investigation in PGX. We also expect that our knowledge of the functional role of genetic variants of this highly polymorphic gene will accompany future discoveries unraveling important biological functions of NQO2.

## Supplementary Materials

The following supporting information can be downloaded at: https://www.mdpi.com/article/10.3390/genes15010087/s1, Table S1: List of characterized *NQO2* SNPs and source sequences.
